# Traditional healers and global surveillance strategies for emerging diseases.

**DOI:** 10.3201/eid0204.960412

**Published:** 1996

**Authors:** N E Groce, M E Reeve


### Traditional Healers and Global Surveillance Strategies for Emerging Diseases: Closing the Gap

A recent position paper by the Centers for Disease Control and Prevention (CDC) stresses that surveillance is critical to an effective defense against new and reemerging infectious diseases and indicates that current international monitoring of such diseases is fragmentary and inadequate (1). Other major studies have also recorded the weaknesses in the present disease reporting system (2-4).

The concept of "global surveillance" implies the coordination of existing networks as well as the addition of state-of-the-art electronic networks to ensure close monitoring of and rapid response to outbreaks, even in the most remote locations (1,2,5-8). Plans for strengthening current surveillance efforts include a global consortium with specialists in epidemiology and infectious diseases working in close collaboration with international agencies, ministries of health, universities, and research laboratories (1,2,6,9-11). Existing programs at the World Health Organization, CDC, the Pan American Health Organization, and elsewhere will be reconfigured to work as a more cohesive system (1). Secure networks will be developed for 1) the transmission of sensitive information; 2) automatic reporting from physicians' offices, hospitals, and laboratories; and 3) the integration of existing and planned information systems. The field application of computer technology, satellite imagery that allows geographically oriented information to be visually and analytically linked to images of the environment, and the development of new statistical and mathematical modeling methods are under discussion (1,3,12).

As medical anthropologists, we note the absence in current plans for global reporting systems of "traditional" or non-Western health care providers, who in communities worldwide are usually the first, and often the only, health specialists to see patients with new or reemerging diseases. These local health specialists, called traditional healers, may have a role to play in the early identification of new or reemerging diseases and could assist in coordinating responses to outbreaks and providing public health education at the local or regional levels.

Most people around the world have little access to modern medical systems (13-15). Even though immunizations and antibiotics increasingly find their way into indigenous systems, healers, midwives, bone setters, herbalists, and other traditional health experts provide most or all medical care. The more remote, indigent, or traditional the population, the greater the likelihood that it will have little access to modern medical care (13,16). If such care is sought, it will be only as a last resort, should traditional healers prove unable to address the illnesses (16) . In many communities, modern medicine is not perceived as better than traditional healing, and it is often more costly. Distance from modern medical resources is another barrier. Medical care that is not sensitive to cultural differences as well as the belief that some types of diseases are not treatable by modern medicine are also prevalent. These beliefs are particularly common in developing countries; however, traditional healers also practice in many ethnic and minority communities in industrialized societies throughout North America, Europe, and Australia (17-19).

A primary dependence on traditional healers continues in areas that, until recently, were considered largely untouched by modern development. It is in just such areas that much of the recent economic development has triggered rapid ecologic change. These once sparsely populated areas, now being pulled into the global economic sphere through logging, mining, and agriculture, are precisely the areas where it is anticipated that many new infectious diseases will originate, as increasing populations come in contact with previously undisturbed vectors of infectious diseases. In such areas, traditional healers are often in a unique position to identify new and reemerging diseases. Whatever their specialty, traditional healers are 1) familiar with diseases commonly found locally; 2) aware of an increase or decrease in the incidence of such diseases in their patient population; 3) among the very first to see cases of new diseases; and 4) cognizant of the recurrence of a disease they have not seen in some time. If traditional healers are not tied into the global reporting network in a systematic and effective manner, their knowledge of new or reemerging disease information may reach the outside world late or in many cases, not at all. Traditional healers differ not only from country to country, but often from region to region and from one ethnic or minority group to the next. An adequate surveillance system must ensure that in each instance the most appropriate traditional healers are included in some type of timely warning system.

Including traditional healers into a global system does not mean that scientists and clinicians must agree with indigenous explanations of the causes or treatments of infectious diseases. Nor does it require that traditional healers accept modern assumptions about the causes, presence, or treatment of such diseases. However, a complete surveillance system does require that participants cooperate and maintain professional respect and courtesy. The goal is a surveillance system that is sensitive to cultural differences and in which new or unusual medical events can be reported quickly and accurately from the traditional healer to the local medical officials in the hospital or laboratory linked to the global surveillance system.

In recommending the inclusion of traditional healers in a global surveillance network, we do not seek to minimize the differences, or the animosity, between these healers and modern medical practitioners (14). Moreover, the relevant strengths and weakness of traditional healing are not the issue here. Critical time, however, may be lost unless all resources are tied into a disease reporting system.

Lines of communication must be established between traditional healers and local health care systems that serve as the "up-links" to the regional, national, and international early warning systems. A system in which traditional healers know whom to contact and how to establish contact quickly is essential. Traditional healers must be taught why, what, when, and how to report unusual symptoms in their patients to local officials. Training for traditional healers must include explaining, (in terms that are culturally relevant to their understanding of illness and health) why scientists outside their communities need timely medical information from their local practices. What to report is of equal concern. Healers must be briefed in what is reportable. A checklist of specific symptoms, such as new or unusual fevers, rashes, or lesions could be developed for reference. Such a checklist could also include questions on the apparent mode of transmission of the disease, (whether it is appearing in members of the same household; in specific parts of a local area, such as households that share a common water source or are located near a forested area; or in sex partners). The development and circulation of a pictorial reference guide of diseases found in an area might facilitate communication between healers and local officials. Specific guidelines should ensure that reporting is done quickly. Finally, a clear and workable reporting system, with specific information about whom to contact at the local level should be established.

An effective surveillance program must include a systematic educational component for local health officials, with specific discussion about the need to include traditional healers, what information these healers are asked to provide, and how this information, once conveyed to local health officials, must be transmitted to the regional hospital, universities, and ministries of health quickly and effectively. Because many local health officials have heavy demands placed on their time, the more straightforward this transmission link is made, the better for all concerned. The local health official is the key "up-link" between the remote field and the regional or national surveillance centers where a more careful and systematic evaluation of the new or reemerging infectious disease should begin.

Finally, training for both healers and those to whom they report must be comprehensive, and its effectiveness must be evaluated often. A communications bridge must be established and maintained if global warning is to be truly effective.

### Acknowledgment

We thank Mark L. Wilson, Infectious Disease Division, Yale School of Public Health, for his helpful comments on this manuscript.

**Nora Ellen Groce* and**
**Mary Elizabeth Reeve†**
*Division of Health Policy, Yale School of Public Health and †Division of International Health, Yale School of Public Health

### References

1. Centers for Disease Control and Prevention. Addressing emerging infectious disease threats: a prevention strategy for the United States. Atlanta, GA: U.S. Department of Health and Human Services, Public Health Service, 1994.
2. Wilson ME, Levins R, Spielman A., editors. Detection, surveillance, and response to emerging diseases. In Disease in evolution: global changes and emergence of infectious diseases. New York Academy of Sciences 1994;70:336-8.
3. World Health Organization. Emerging infectious diseases: Memorandum from a WHO meeting. Bull World Health Organ 1994;72:845-50.
4. Hughes J. Conference on “Emerging infectious diseases: meeting the challenge.” Emerging Infectious Diseases 1995;1:101.
5. Bartlett C, Gill N. International surveillance of disease. Lancet 1993;341:1003-6.
6. Henderson D. Surveillance systems and intergovernmental cooperation. In: More, SS, editor. Emerging Viruses. New York: Oxford University Press, 1993.
7. Marwick C. Effective response to emerging diseases called an essential priority worldwide. JAMA 1995;273:189-90.
8. Institute of Medicine. Emerging infections, microbial threats to health in the United States. Washington, DC: National Academy Press, 1992.
9. Pan American Health Organization. Combating emerging infectious diseases. Washington, DC: PAHO, June 1995.
10. LeDuc J, Tikhomirov E. Global surveillance for recognition and response to emerging diseases. New York Academy of Sciences. 1994;740:341-5.
11. O'Brien T, Stelling J. WHONET: An information system for monitoring antimicrobial resistance. Emerging Infectious Diseases 1995;1:66.
12. Vacalis T, Bartlett C, Shapiro C. Electronic communication and the future of international public health surveillance. Emerging Infectious Diseases 1995;1:34-5.
13. Foster G, Anderson B. Medical Anthropology. New York: Knopf, 1978.
14. Velimirovic B. Is integration of traditional and western medicine really possible? In: Coriel J, Mull JD, editors. Anthropology and Primary Health Care. Boulder, CO: Westview Press 1990.
15. Gumede MV. Traditional healers: a medical practitioner's perspective. Braamfontein: Skotaville Publishers, 1990.
16. Pillsbury B. Policy and evaluation perspectives on traditional health practitioners in national health care systems. Social Science and Medicine 1982;16:1825-34.
17. Galanti GA. Caring for Patients from Different Cultures Philadelphia: University of Pennsylvania Press, 1991.
18. Lynch E, Hanson M. Developing cross-cultural competence. Baltimore: Paul Brooks 1992.
19. Brown K. Mama Lola: A Vodou Priestess in Brooklyn. Berkeley: University of California Press, 1991.

